# Recruitment and retention of low-income minority women in a behavioral intervention to reduce smoking, depression, and intimate partner violence during pregnancy

**DOI:** 10.1186/1471-2458-7-233

**Published:** 2007-09-06

**Authors:** M Nabil El-Khorazaty, Allan A Johnson, Michele Kiely, Ayman AE El-Mohandes, Siva Subramanian, Haziel A Laryea, Kennan B Murray, Jutta S Thornberry, Jill G Joseph

**Affiliations:** 1 Statistics and Epidemiology Unit, RTI International, 6110 Executive Blvd., Suite 902, Rockville, MD 20852-3903, US; 2 Division of Allied Health Sciences, College of Pharmacy, Nursing and Allied Health Sciences, Howard University, Washington, DC 20059, US; 3 Collaborative Studies Unit, DESPR/NICHD/NIH, 6100 Executive Blvd., Rm 7B05, Rockville, MD 20852-7510, US; 4 Department of Prevention and Community Health, School of Public Health and Health Services, The George Washington University Medical Center, 2175 K Street, NW, Washington, DC 20037, US; 5 Division of Neonatology, Georgetown University Hospital, 3800 Reservoir Road NW, Washington D.C. 20007, US; 6 c/o Allan A. Johnson, Division of Allied Health Sciences, College of Pharmacy, Nursing and Allied Health Sciences, Howard University, Washington, DC 20059, US; 7 6537 Ashby Grove Loop, Haymarket, VA 20169; 8 Statistics and Epidemiology Unit, Research Triangle InstituteInternational, 6110 Executive Blvd., Suite 902, Rockville, MD 20852-3903, US; 9 Center for Health Services and Community Research, Children's National Medical Center, 111 Michigan Avenue, NW, Washington, DC 20010, US

## Abstract

**Background:**

Researchers have frequently encountered difficulties in the recruitment and retention of minorities resulting in their under-representation in clinical trials. This report describes the successful strategies of recruitment and retention of African Americans and Latinos in a randomized clinical trial to reduce smoking, depression and intimate partner violence during pregnancy. Socio-demographic characteristics and risk profiles of retained vs. non-retained women and lost to follow-up vs. dropped-out women are presented. In addition, subgroups of pregnant women who are less (more) likely to be retained are identified.

**Methods:**

Pregnant African American women and Latinas who were Washington, DC residents, aged 18 years or more, and of 28 weeks gestational age or less were recruited at six prenatal care clinics. Potentially eligible women were screened for socio-demographic eligibility and the presence of the selected behavioral and psychological risks using an Audio Computer-Assisted Self-Interview. Eligible women who consented to participate completed a baseline telephone evaluation after which they were enrolled in the study and randomly assigned to either the intervention or the usual care group.

**Results:**

Of the 1,398 eligible women, 1,191 (85%) agreed to participate in the study. Of the 1,191 women agreeing to participate, 1,070 completed the baseline evaluation and were enrolled in the study and randomized, for a recruitment rate of 90%. Of those enrolled, 1,044 were African American women. A total of 849 women completed the study, for a retention rate of 79%. Five percent dropped out and 12% were lost-to-follow up. Women retained in the study and those not retained were not statistically different with regard to socio-demographic characteristics and the targeted risks. Retention strategies included financial and other incentives, regular updates of contact information which was tracked and monitored by a computerized data management system available to all project staff, and attention to cultural competence with implementation of study procedures by appropriately selected, trained, and supervised staff. Single, less educated, alcohol and drug users, non-working, and non-WIC women represent minority women with expected low retention rates.

**Conclusion:**

We conclude that with targeted recruitment and retention strategies, minority women will participate at high rates in behavioral clinical trials. We also found that women who drop out are different from women who are lost to follow-up, and require different strategies to optimize their completion of the study.

## Background

There is a long standing concern that minorities, in particular African Americans and Hispanics, are less willing to participate in clinical research than non-minorities. This belief exists not only among researchers [1] but among the minority populations as well. Many individuals believe that the Tuskegee Syphilis Study has negatively influenced the relationship between African Americans and the biomedical research community [2,3]. However, Gamble [4] suggested that this distrust predated the public revelations about the Tuskegee study. When the federal government mandated in the 1993 Revitalization Act the proportional inclusion of all racial/ethnic minorities in human subject research, factors affecting recruitment and retention of minorities became a topic of more intense discussion. The scientific community increasingly recognizes the motivation behind federally mandated inclusion criteria: insuring that the results of research benefit all groups. To achieve this, it is necessary to guarantee that the results of medical and behavioral research are applicable and available to all members of the population. Also, it is important to determine whether the results differ for a specific race and/or ethnic group, so that generalization from one race/ethnic group to another is valid.

There is a broad literature exploring reasons for differential minority participation in clinical trials. In addition to the memory of the Tuskegee Study [2,5], there is a more generalized distrust among minorities of the medical community [6-9] and a history of negative experiences with health facilities [6]. Other reasons cited include those related to the organization and communication of research and those related to circumstances in minority communities. Issues related to the organization and communication include the paucity of clinical trials occurring at institutions where minorities seek medical care [2,7], ineffective communication by research staff [7,8], complex study medicine regimens, difficulty in rescheduling appointments due to lack of flexibility on the part of study personnel [3,10], complicated and cumbersome record-keeping requirements [11,12], lack of feedback, and ineffective informed consent procedures [6,10]. On the other hand, issues related to the circumstances of minority populations include fear of being used as guinea pigs [8,13], lack of awareness of medical trials [8,10], the influences of relatives and friends, working multiple jobs, conflicting work schedules [6,10], barriers to transportation [7,10] and child care, poor incentives, inability to complete self-administered forms (i.e., low literacy), language barriers [8,14], personal reasons, lack of time, and other priorities which take precedence over study obligations [11,12]. Other problems mentioned more often by ethnically diverse subjects who withdrew from a multicenter randomized controlled research trial were lack of time, negative side effects, and dissatisfaction with the overall research process [3,10].

Beliefs that minorities are less willing to participate in research, when compared to non-minority populations, stem mainly from their under-representation in various surveys due to higher non-response rates and/or lack of access to health research. An accurate validation of these beliefs to evaluate minority recruitment into many clinical and behavioral intervention studies is not possible at all times. Unlike response rates in surveys, lack of reporting of the numbers of screened/potential/eligible subjects in clinical and behavioral research necessary for the calculation of recruitment rates by race and ethnicity makes it impossible to assess the success/failure of minority recruitment [11]. A recent report by Wendler, et al. [15] suggests, contrary to popular belief, that the differences in the rates at which non-Hispanic whites and minorities agree to participate in health research are small. This conclusion is based on a review of 20 health research studies (3 interview studies, 10 clinical intervention studies, and 7 surgery trials) that included sufficient data to determine consent rates by race or ethnicity. Where differences (although non-significant) did occur, minority groups tended to be more, not less, willing to participate in research. This review suggests that the primary reasons for lack of participation by minority groups is the failure to invite them to participate, to provide accessible sites for participation, and failure to overcome barriers such as childcare arrangements and transportation costs. Because this analysis focused on clinical intervention trials and did not include behavioral or prevention trials or natural history studies, it is possible that the study characteristics may more strongly affect participation than race or ethnicity. For example, in a behavioral and counseling educational intervention, it is possible that the inability to foresee immediate and/or personal benefit for the time commitment that is required may discourage participation. This would suggest that successful recruitment and retention of minorities may depend also on the type of research that is being conducted.

This paper focuses on the recruitment and retention of participants in a behavioral and counseling intervention trial entitled "Interventions for Risk Factors in Pregnant Women in Washington, DC: An Integrated Approach" or DC-Healthy Outcomes of Pregnancy Education (hereinafter referred to as Project DC-HOPE). In an attempt to understand why our recruitment and retention strategies were so successful, the socio-demographic characteristics and risk profiles of women who were retained are compared to those who were not retained. Since outcome measures regarding risk factors may be evaluated at two time points (at delivery and at 8–10 weeks postpartum), retention can be measured at these two distinct points. Retention rates, as estimated on the basis of completed interviews, are compared for follow-up interviewing prior to delivery and at the postpartum period by socio-demographic and risk characteristics. Comparisons of those who completed these interviews with those who did not complete these interviews are also presented.

## Methods

Project DC-HOPE was one project within the NIH-DC Initiative to Reduce Infant Mortality in Minority Populations in the District of Columbia, a collaborative effort involving the Children's National Medical Center, George Washington University, Georgetown University, Howard University, the National Institute of Child Health and Human Development (NICHD)/NIH/DHHS, and RTI International. Project DC-HOPE was a randomized clinical trial targeting smoking (including environmental tobacco smoke exposure), depression, and intimate partner violence (IPV). The primary goal was to estimate whether a multi-modal, integrated counseling and educational intervention reduces smoking and environmental tobacco smoke exposure (ETSE), depression, and IPV (defined as being victimized) among pregnant African American and Latina women. A secondary goal was to estimate whether a clinic-based intervention reducing smoking, ETSE, depression, and IPV in pregnancy would reduce adverse pregnancy outcomes (e.g., prematurity and low birth weight) and lower infant morbidity and mortality.

Outcome measures were selected to address both sets of goals. Medical records of infants and mothers were abstracted to obtain data on pregnancy outcome measures. In addition, an assessment battery of standardized and validated self-report measures of the targeted risks and cotinine levels based on saliva specimens were collected. These assessments were obtained at baseline, 22–26 weeks and 34–38 weeks of gestation, and 8–10 weeks postpartum. Subjects were recruited at six prenatal care clinics in Washington, DC. Women were eligible to participate if they were Washington, D.C. residents, African American or Latino, at least 18 years of age, at 28 weeks gestation or less, and English speaking. These eligibility criteria were verified from clinic records. Institutional review boards at Howard University, RTI International, and NIH approved the study.

To ensure adequate statistical power for testing the hypotheses that multidisciplinary integrated intervention will result in reductions in targeted risk factors and improvements in pregnancy outcomes, determination of sample size requirements was essential. Based on the number of births to African American women in Washington, DC, estimates of the number of women appearing at participating clinics in their first two trimesters of pregnancy, the prevalence of the risk factors estimated from previous studies, the estimated refusal rate, and the 2-year recruitment period, a total of 1,750 pregnant women were expected to enroll. Because of the study design and eligibility criteria, participants identified would have a 100% prevalence of a specific risk factor. Assuming a 20% drop-out rate, and 20% loss to follow-up, 1,050 women were expected to be retained at the end of the follow-up period (525 in each of the care groups). This number, assuming a 5% level of significance, 80% power, would allow the detection of 10–20% reductions in risk-specific factors among those in the intervention group from a 100% prevalence at recruitment time. This number was also sufficient to detect a 25% reduction in prematurity and low birthweight combined in the intervention group as compared to that for the usual care (estimated at 20%).

Project DC-HOPE staff members were grouped into four functional teams: Recruitment Specialists (RSs) conducted participant recruitment and retention of study subjects and collected saliva specimens; Pregnancy Advisors (PAs) delivered the intervention; telephone interviewers administered the evaluation interviews; and abstractors obtained information from medical records.

Recruitment took place between July 9, 2001 and October 31, 2003, four months beyond the original planned 2-year period. Follow-up activities of participants continued until July 31, 2004. The project started at four clinic sites. Because one site closed ten months after the start of the study, a fifth site was added in February 2002, and an additional sixth site was added in May 2003. These steps were taken to ensure the recruitment of the required sample size.

RSs approached women presenting for prenatal care while waiting for their appointments. Clinic logs were monitored closely on a daily basis for both returned participants and new clients. The RSs briefly explained the objectives of the study to the women and provided them with a brochure describing the study and answers to common questions. Interested women gave written consent for screening. Women were screened for socio-demographic eligibility and the presence of one or more of the four targeted risks using an Audio-Computer-Assisted Self Interview (A-CASI). A-CASI methodology allows subjects to listen to digitally recorded questions on headphones that are connected to a laptop computer while the question is simultaneously displayed on the computer screen. As a response choice is heard, it is highlighted on the screen. The subject answers by touching the chosen response option on the screen. A-CASI offers an environment in which sensitive information is easily and privately reported without the need for special computer skills or reading ability. Consequently, risky behaviors are reported more frequently than in more conventional interviewing or self-administered paper and pencil forms [16-19].

The recruitment process was flexible in order to enhance participation. If a woman were reluctant to start the screening process due to time constraints, the RSs would suggest that she complete the screening and recruitment process when she returned to the clinic at a later date.

Women found to be eligible based on A-CASI responses were invited to participate in the study and provided with all information necessary to make an informed and knowledgeable decision. Those interested signed a second form consenting to participate in the randomized trial once they completed the baseline interview.

These consenting participants were asked to provide contact information, including the best phone number for reaching them and the names and telephone numbers of two relatives or friends who did not live with them. Efforts were taken to develop contact procedures from the research staff who maintained confidentiality when communicating with participants outside the clinic setting. Addresses were collected to facilitate tracing efforts, but the women were informed that they would not receive mail from Project DC-HOPE. Since one of the screened risk factors was IPV, staff did not want to raise women's risk for abuse by receiving mail from the study that might be negatively regarded by an abusive partner, or would expose her pregnancy. For similar reasons, women were asked whether or not telephone messages from Project DC-HOPE staff could be left on their telephone answering machines. If not, this was noted in her computerized record accessible by all project teams.

After completing the baseline interview, women were randomly assigned to usual care or intervention group. Site- and risk-specific block randomization of participants was conducted. Women randomized to the intervention group met with trained PAs (in addition to seeing their physician) either immediately before or after a routine prenatal visit and at two postpartum sessions during which they received individualized counseling targeting their area(s) of risk. Women in the intervention group attended on average 3.9 ± 2.8 prenatal sessions, and 53.6% attended at least 4 prenatal sessions. Participants spent an average of 35.3 ± 14.8 minutes per session. Women assigned to the usual care group met with their primary care providers as per standard clinic practice.

### Recruitment and retention strategies

Several strategies were implemented in Project DC-HOPE which were designed to promote successful recruitment and retention of study participants. These strategies received careful consideration, taking into account the goals of the study and the type and duration of the intervention. This is important in such a community-based research study in which reliance on clinic infrastructure and personnel influences the success of the study. The strategies included features of the study design, consistent contact with study participants, financial incentives, recruitment training, cooperation from clinic staff, effective tracking of study participants, and continuous monitoring of study progress. A description of each of these elements follows.

As part of the study design, women had to complete the baseline interview before they were randomized and enrolled into the study. Consequently, women were identified who were more likely to complete the study, since there were several opportunities (completing the A-CASI screening, providing main study consent, and completing the baseline interview) leading up to randomization during which they could refuse or be lost to follow-up. This strategy also preserved the balance between the usual care and intervention groups in order to facilitate the intent-to-treat analysis of the impact of the intervention. In fact, the difference in the retention rate between the intervention group (78%) and the usual care group (81%) was not significantly different. A second feature of the study design, which contributed to high retention by reducing participant burden, was scheduling all in-person study activities to coincide with prenatal care visits. This included the collection of saliva, the dispensation of incentives, and the delivery of the intervention sessions.

Critical to retention and the success of a longitudinal study is consistent contact with the study participants and updated contact information [20,21]. For Project DC-HOPE, RSs maintained frequent telephone contacts with the participants to remind them of intervention sessions and to reschedule missed appointments. Contact information was updated each time project staff had personal contact with study participants, including when monetary incentives were dispensed and saliva samples were collected, and at each intervention session. Contact information was also updated at the completion of each telephone interview. The four project teams maintained detailed documentation of the time and day of attempted phone calls (both successful and unsuccessful) to determine the best time to reach the participants. Senturia, et al. [21] found that timing of phone contacts and their intensity are crucial in retaining participants. Thus, great efforts were taken to develop contact procedures for the research staff to maintain confidentiality when communicating with participants outside the clinic setting. In addition, a concerted effort was made by study staff to track hard-to-find participants. The contact information database was checked to obtain the most up-to-date locator information. The subject was then telephoned at her primary and (where necessary) her secondary telephone numbers. If the subject were not reached, Directory Assistance was consulted for a new telephone listing. If a new listing were not found, the clinic staff was consulted for updated or different contact information. In addition, the planned hospital delivery site was contacted to determine if the subject had been admitted.

Financial incentives to compensate participants for their time and effort also contributed to successful recruitment and retention of study participants. All women received $5 for completing the A-CASI screening, a 30-minute telephone card for providing main study consent, and $15 for each telephone interview. Women in the intervention group received $10 for each intervention session and additional $15 and $25 gift certificates for the first and second postpartum intervention sessions, respectively.

The efforts of the RSs and the extensive training they received contributed to successful recruitment. Mostly African American and females, the RSs learned that their verbal and non-verbal behavior was an important part of obtaining cooperation and promoting participant confidence and trust. Rapport was imperative for successful recruitment and was achieved through showing sensitivity to the women and their experiences. Specifically, RSs were taught to be:

• alert, clear spoken, and good listeners;

• positive and assertive, but not aggressive;

• responsive to the woman's reasons for reluctance;

• respectful and culturally sensitive;

• confident, sincere, and spontaneous in their introduction; and

• credible, by knowing the objectives of Project DC-HOPE and the activities required for participation.

Training involved role-playing to address different situations and types of women. Training of new RSs also included the sharing of experiences by the veteran RSs. In addition, RSs discussed recruitment strategy ideas in periodic meetings with the supervisor of data collection and principal investigators.

Cooperation from the clinic staff was another critical strategy for the recruitment and retention efforts. As suggested by Wiemann and colleagues [22] this [clinic staff] cooperation is facilitated by educating them on the study design and designing the project implementation procedures with the least amount of interference with the daily functioning of the clinic. Before launching the study, the Principal Investigators held meetings with the clinic staff to explain the purpose of the study and describe their role in the study. Their understanding of the study design and sense of involvement with the recruitment and retention process helped maintain the integrity of the research effort and provide the project easy access to the clinic sites. Throughout the duration of the study, project supervisory staff made frequent visits to clinic sites to address problems and to demonstrate willingness to be flexible and modify procedures to adapt to the individual clinic needs. The study also provided partial salary support for the clinic staff who were assuming the extra responsibility of collaborating with the RSs and PAs.

A Data Management System (DMS) was developed to share and track information about study participants by the four functional project teams. The DMS was a PC-based computerized system that allowed for the collection, aggregation, and reporting of the study data. The DMS tracked upcoming events (visits, interviews, incentives, intervention, etc.) for individual study participants and was available to all project teams on computers dedicated to the study at each clinic site, interviewing site, and medical record abstraction site. Reports generated by the DMS made it possible for the project teams to prepare for upcoming events, such as a participant's prenatal care visit or intervention session, or the need to collect a saliva sample or dispense an incentive payment, and were critical in the recruitment and retention efforts. Types of reports included:

• a list of patients previously seen at the clinic with their recruitment status,

• a list of scheduled appointments for a given day with required activities,

• a list of participants with a specific activity due or overdue,

• a report for each study participant summarizing their completed study activities, along with personal information such as contact information, their next prenatal care visit, and pregnancy status.

The Data Coordinating Center (DCC) staff responsible for the implementation of the DMS maintained close communications with the functional project teams, provided quick response to any issues, and immediately resolved any problems the staff had with the system. Senturia, et al. [21] recommended such a strategy as "essential to achieving nearly complete follow-up within a population historically difficult to follow."

Recruitment and enrollment status reports, first weekly, then bi-weekly, and finally monthly, were used to monitor study progress. These reports provided site-specific information for all study activities, beginning with the number of women approached and screened. In addition to the number of eligible and consenting women, reasons for ineligibility and refusals were also reported. Actions to address recruitment issues were taken accordingly. For women enrolled in the study and randomized, the reports provided the number of women who withdrew from the study or were lost to follow-up. Number of completed intervention sessions, completed telephone interviews for each evaluation time interval, and completed medical record abstractions were also reported. These data provided the study management staff with information at each stage of the study and identified potential problems so that appropriate actions could be taken in a timely fashion, such as the addition of clinic sites to increase recruitment. Site-to-site variability in recruitment was monitored and re-training or increased supervision provided as needed.

### Statistical analysis

The statistical analyses of recruitment and retention data proceeded in three stages. First, we present various screening, eligibility, and enrollment results. Differentials in retention rates, comparing socio-demographic characteristics and risk profiles of women retained with those women not retained, were investigated using Fisher's exact tests and Chi-square tests for categorical variables. The differences in means for continuous variables were tested using t-tests. Retention rates at delivery and at postpartum were computed. Differentials in sample characteristics by clinic site (not shown here) were evaluated. The results indicated that socio-demographic characteristics of participants differed across sites and thus needed to be and were accounted for in the analysis.

In the second stage, we investigated multivariable relationships to identify the various socio-demographic characteristics that contributed to differentials in retention/attrition rates, after controlling for all factors using logistic regression analysis [23]. A multivariable model is presented with various sets of predictors: (1) socio-demographic characteristics, (2) clinic sites, (3) risk factors at baseline interview, and (4) risk predictors. This model includes fourteen predictors. The number of women not completing the postpartum interview, when divided by the number of predictors is approximately 15. This number is greater than the recommended minimum number of events per predictor. This assures the reliability of the estimates of the regression coefficients and the validity of the statistical inference based on this model [24].

In the third stage, we used Classification and Regression Trees (CART) methodology to identify the characteristics of various homogeneous subgroups of women who were likely not to be retained. CART methodology [25,26] helped formulate distinct groups of retained and not retained women, thus identifying those characteristics predicting retention status. CART methodology is a non-parametric exploratory procedure that does not require the stringent assumptions of other parametric statistical methods and tests. For more details, the reader may consult various cited references [27-30].

## Results

### Recruitment and participation status

**Figure 1:**
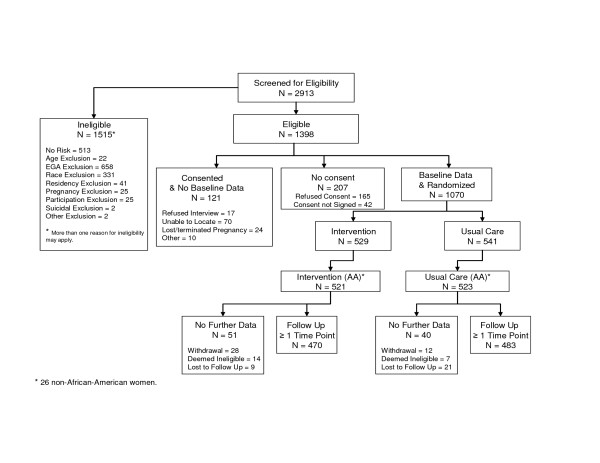
Screening, Eligibility, Recruitment and Retention: Project DC-HOPE.

A total of 4,213 women were approached for A-CASI screening at the six clinic sites. Of these women, 649 refused and 651 never completed the A-CASI screening to determine their eligibility. As shown in Figure 1, the remaining 2,913 women were screened for demographic and risk eligibility. Forty-eight percent (1,398 women) were eligible for recruitment. Of the 1,515 who were ineligible, 919 women did not meet the demographic eligibility criteria, 513 did not report any of the designated risks, 81 failed the eligibility verification (conducted later on the basis of the clinic records), and two were found to be potentially suicidal.

Eligibility rates per site were significantly different. As noted in Table 1, only 54% and 63% of those women appearing at clinic sites C and E, respectively, for prenatal care visits met the demographic eligibility criteria, as compared to 72% to 84% for the other four sites. The main demographic reason for ineligibility in five of the six sites was gestational age of more than 28 weeks (ranging from 77% to 94%), and in the sixth site 64% did not meet the race/ethnicity criteria.

**Table 1:** Screening, eligibility, consenting, and enrollment by clinic site

**Clinic site**	**Number completed A-CASI**	**Number demographically eligible**		Number eligible for recruitment**		**Number consented**		**No. completed baseline/enrolled and randomized**		**Enrolled as % of eligible**
		
	**N**	**N**	%*	**N**	%*	**N**	%*	**N**	%*	
**A**	322	269	83.5	217	67.4	198	91.2	178	89.9	82.0
**B**	237	189	79.7	133	56.1	118	88.7	106	89.8	79.7
**C**	956	518	54.2	320	33.5	220	68.8	208	94.5	65.0
**D**	450	365	81.1	259	57.6	232	89.6	197	84.9	76.1
**E**	331	210	63.4	135	40.8	112	83.0	108	96.4	80.0
**F**	617	443	71.8	334	54.1	311	93.1	273	87.8	81.7
**TOTAL**	2913	1994	68.5	1389	47.7	1191	85.7	1070	89.8	77.0

* As percentages of the number in the previous category.

** Women demographically eligible and were identified by at least one of the three risk factors.

Of the 1,398 eligible women, 85% (1,191 women) consented to participate in the study (165 refused to sign the main consent of the study and 42 were not offered the consent for logistic reasons). Again, consented rates out of those eligible for the study by site were significantly different. For those 165 women who refused to consent, the most common reasons for refusing to sign the main consent were denial of need for help (32%), lack of interest (29%), claims to be beyond 28 weeks of gestation (16%), and do not have enough time (6%). Of note, only 3% of those refusing gave study intrusiveness as the reason.

Of the 1,191 women consenting to participate, 90% (1,070 women) completed the baseline telephone interview and were randomized into the study. Of the 121 women who consented but did not complete the baseline interview and thus not enrolled in the study, 70 were not located, 24 either lost or terminated their pregnancy, 17 refused the baseline interview, and 10 were not enrolled due to other reasons.

Enrolled women were, on average, 24.6 years old and reported that they were 17.1 weeks of gestation at recruitment. The majority were Black or African-American (98%) and single, separated, widowed or divorced (76%). Nearly two-thirds of the women had other children (68%), were not working (63%), and more than three-fourths received Medicaid (77%).

### Retention status

Of the 1,070 enrolled women, 21% (221 women) discontinued the study before completing the postpartum telephone interview. Five percent (58 women) dropped out, 12% (128 women) were lost to follow-up, and 3% (35 women) did not complete the study because they were identified as ineligible after they were enrolled in the study. Eight of these ineligible women were potentially suicidal, five had previously participated in the study, ten were found to have gestational age greater than 28 weeks at recruitment, three terminated their pregnancy, one delivered before the baseline interview was administered, and the remaining 18 women were deemed ineligible for other reasons, including age, pregnancy status, and significant medical co-morbidities. The remaining 849 completed the final postpartum interview, for a retention rate of 79%. The reasons for those 58 who dropped out included loss of interest in the study (40%), moving out of Washington, DC (11%), denial of risk (11%), lack of time (7%), dislike of questions asked (5%), miscarriages and abortions (5%), denial of need for the intervention (4%), and illness (4%).

As reported earlier, 98% (1,044) of the 1,070 enrolled women were African American. Of these 1,044 African American women, 91.3% (953 women) completed at least one of the follow-up interviews. (See Figure 1.) Only 4% of the African American women (40) withdrew from the study, 3% (30) were lost to follow-up, and 2% (21) were deemed ineligible after enrollment. A total of 831 African American women (80%) completed the final postpartum interview.

### Comparison of characteristics between retained and not retained participants

We compared the socio-demographic (measured at baseline) and risk characteristics between women who were retained (completed the study per protocol) and women who were not retained because they either dropped out or were lost to follow-up. Chi-square and t test statistics for differences were performed. As shown in Table 2, women who were retained were slightly older (24.6 vs. 24.3 years), better educated, married, and employed. However, no significant differences were found between the two care groups.

**Table 2:** Socio-demographic and risk characteristics by retention status

**Socio-demographic & risk characteristics**	**Not retained**	**Retained**	**Significance level**
**Socio-demographic characteristics**			
**Maternal age**	24.3 ± 5.4	24.6 ± 5.4	0.42
**Educational level:**			
Below high school	76 (34.5%)	245 (28.8%)	0.25
High school graduate/GED	97 (44.1%)	399 (46.9%)	
Some college	47 (21.4%)	206 (24.2%)	
**Relationship status:**			
Single/separated/widowed/divorced	179 (81.4%)	637 (74.9%)	0.05
Married/Living with a partner	41 (18.6%)	213 (25.1%)	
**Employed**	72 (32.7%)	323 (38.0%)	0.16
**Receives Medicaid**	171 (78.1%)	653 (77.2%)	0.86
**Gestational age at recruitment (weeks)**	17.2 ± 7.4	17.1 ± 6.6	0.75
**Alcohol use during pregnancy**	46 (20.9%)	183 (21.5%)	0.93
**Drug use during pregnancy**	36 (16.4%)	151 (17.8%)	0.69
**A-CASI screening risk results**			
**Active smoking**	118 (53.6%)	396 (46.6%)	0.07
**Environmental tobacco smoking (ETSE)**	175 (79.6%)	710 (83.5%)	0.16
**Depression**	78 (35.4%)	308 (36.2%)	0.87
**Intimate partner violence (IPV)**	46 (20.9%)	178 (20.9%)	1.00

Table 3 shows comparisons between women who dropped out or withdrew from the study to those who were lost to follow-up. Women in these two types of non-retention are not significantly different with respect to risk prevalence. However, women who dropped-out are significantly older (25.9 vs. 23.3 years, *p *< 0.01), less educated (*p *= 0.01), less likely to be single, separated, widowed, or divorced (47% vs. 87%, *p *< 0.01), more likely to be working (47% vs. 31%, *p *= 0.04), and less likely to be receiving Medicaid (66% vs. 84%, *p *< 0.01). Interestingly, the overall non-retention rate of the intervention group and usual care group is the same (about 17%); 90 versus 89 participants, respectively. However, non-retained women by type of retention are significantly different. Women in the intervention group were more likely to drop out or withdraw from the study, than those in the usual care group (69% vs. 31%, *p *< 0.01). Women in the usual care group were more likely to be lost to follow-up as compared to the intervention group (58% vs. 42%).

**Table 3:** Socio-demographic and Risk Characteristics by Types of Non-Retention

**Socio-Demographic & Risk Characteristics**	**Lost-to-follow-up**	**Dropped-out/withdrew**	**Significance Level**
**Socio-demographic characteristics**			
**Maternal age**	23.3 ± 5.0	25.9 ± 5.3	< 0.01
**Educational level:**			
Below high school	43 (34.7%)	15 (27.3%)	0.01
High school graduate/GED	62 (50.0%)	21 (38.2%)	
Some college	19 (15.3%)	19 (34.5%)	
**Relationship status:**			
Single/separated/widowed/divorced	108 (87.1%)	26 (47.3%)	< 0.01
Married/Living with a partner	16 (12.9%)	29 (52.7%)	
**Employed**	38 (30.6%)	26 (47.3%)	0.04
**Number of Children < 18 living with her**	1.31 ± 1.32	1.33 ± 1.41	0.93
**Receives medicaid**	104 (83.9%)	36 (65.5%)	< 0.01
**Gestational age at recruitment (weeks)**	19.4 ± 7.1	18.5 ± 6.7	0.42
**Alcohol use during pregnancy**	24 (19.4%)	13 (23.6%)	0.55
**Drug use during pregnancy**	17 (13.7%)	10 (18.2%)	0.50
**A-CASI screening risk results**			
**Active smoking**	25 (20.2%)	8 (14.5%)	0.41
**Environmental tobacco smoking (ETSE)**	96 (77.4%)	38 (69.1%)	0.26
**Depression**	59 (47.6%)	19 (34.5%)	0.14
**Intimate partner violence (IPV)**	37 (29.8%)	15 (27.3%)	0.86
**Baseline interview risk results**			
**Active smoking**	60 (48.4%)	36 (65.5%)	0.04
**Environmental tobacco smoking (ETSE)**	105 (84.7%)	42 (76.4%)	0.21
**Depression**	48 (38.7%)	14 (25.5%)	0.09
**Intimate partner violence (IPV)**	31 (25.0%)	7 (12.7%)	0.08
**Care group**			
**Intervention**	52 (41.9%)	38 (69.1%)	< 0.01
**Usual care**	72 (58.1%)	17 (30.9%)	

Comparisons of women in each of these two types of non-retention to those retained (Tables 2 and 3) indicated that women who were lost to follow-up were significantly different from those retained with respect to age, relationship status, and gestational age at recruitment. Women lost to follow-up were younger, less likely to be married or living with a partner, and at a more advanced gestational age at recruitment. On the other hand, women who dropped out were significantly different from those retained only with respect to relationship status. Women who dropped out were more likely to be married or living with a partner. For women who were not retained, neither group is significantly different from those retained with respect to risk prevalence, except for the active smoking risk. Women who dropped-out were more likely to be smokers than women who were retained (66% vs. 47%, *p *= 0.01).

### Completion rates of follow-up interviews

Realizing that prenatal risk behavior could be different from postpartum behavior and that the factors which operate on these two sets of outcomes are different, Project DC-HOPE investigators examined the effects of the intervention at two points in time, at delivery and at 8–10 weeks postpartum. Consequently, retention of the study participants through the prenatal period to complete the prenatal follow-up interviews (retention at delivery) became as important as retention through the postpartum interview (retention at postpartum). In this section, we address the differentials in socio-demographic and risk factors for non-retention at delivery (i.e., women who did not complete the follow-up interview prior to delivery, whether the second or the first interview close to delivery) and compared these factors to non-retention at postpartum (i.e., women who did not complete the final postpartum interview). In order for the comparisons to reflect these differentials with respect to a homogeneous group of minority women, the results in the rest of the paper are limited only to African American women in the study (1,044 women). This is also justified due to the exclusion of a very small number of Hispanic women enrolled (n = 22) and 4 women who failed to complete the baseline interview prior to the termination of their pregnancy.

Differentials of non-completion rates of interviews by socio-demographic characteristics and clinic sites among African American women are presented in Table 4. Women who were not receiving WIC support were less likely to complete a follow-up interview (45% non-completion of a follow-up interview prior to delivery and 47% non-completion of the final postpartum interview). This is about 20–24% higher than the next highest non-completion rates. Meanwhile, women receiving WIC achieved the highest interview completion rates (87% and 85%, for retention at delivery and at postpartum, respectively). Other non-completion rates for various socio-demographic groups were in the 15–16% range. There was also a tendency for non-completion rates of interviews to decrease with education, for those with a high school diploma or more. Retention rates by clinic site ranged from 77.2% to 86.5% at delivery time and from 65.4% to 86.6% at postpartum.

**Table 4:** Comparison of non-completion of follow-up and postpartum interviews by African American participants by background characteristics

**Background characteristics at baseline interview**	**Number eligible & completed baseline**	Not completed a follow-up interview prior to delivery*		**Not completed the postpartum final interview**	
		
		**N**	%**	**N**	%**
**Maternal age:**					
18–22	445	70	15.7	92	20.7
23–27	327	56	17.1	72	22.0
28 +	270	67	24.8	48	17.8
**Gestational age at baseline:**					
< 14 weeks	353	67	19.0	70	19.8
14 + weeks	691	127	18.4	143	20.7
**Educational Level:**					
< High school	316	65	20.6	75	23.7
HS graduate/GED	486	84	17.3	96	19.8
At least some college	242	45	18.6	42	17.4
**Relationship Status:**					
Single/separated/widowed/divorced	797	147	18.4	174	21.8
Married or living with partner	247	47	19.0	39	15.8
**Current work status:**					
Working (full- or part-time)	381	71	18.6	70	18.4
Not currently working	662	123	18.6	143	21.6
**No. children < 18 living with Her:**					
< 4 children	977	182	18.6	192	19.7
4+ children	67	12	17.9	21	31.3
**Medicaid status:**					
Yes	810	156	19.3	166	20.5
No	229	36	15.7	46	20.1
**WIC status:**					
Yes	860	112	13.0	126	14.7
No	184	82	44.6	87	47.3
**Other public assistance:**					
Yes	625	118	18.9	129	20.6
No	416	76	18.3	84	20.2
**Clinic sites:**					
A	177	27	15.3	29	16.4
B	104	14	13.5	36	34.6
C	201	39	19.4	27	13.4
D	191	33	17.3	34	17.8
E	101	23	22.8	23	22.8
F	270	58	21.5	64	23.7
**Care group:**					
Intervention	521	98	18.8	114	21.9
Usual care	523	96	18.4	99	18.9
**Total**	1044	194	18.6	213	20.4

* Completed the second follow-up interview prior to delivery or the first follow-up interview and delivered prior to the time to administer the second follow-up interview.

** Percentages are calculated of the total number of eligible women who completed the baseline interview (second column).

Risk factors' differentials of non-completion rates are addressed in Table 5. While risks were reported during the A-CASI screening for the purposes of randomization and intervention, women were contacted for baseline telephone interview on average 3–4 weeks after screening using more elaborate sets of questions and/or instruments to assess risks. As a result, some women who reported risk(s) at screening were found to have no risk at the time of the baseline interview. Risk factors reported at baseline interview appeared to more significantly impact interview non-completion rates. Women with any of the four risks factors addressed in this study showed higher non-completion rates than those with none of the four risks (19% vs. 14% for not completing a follow-up interview prior to delivery and 21% vs. 17% for not completing the final postpartum interview). Pregnant women who were actively smoking showed higher non-completion rates than any group of women with other risk factors. Interestingly, women with only IPV risk had the lowest non-completion rates. For the postpartum interview, women with depression risk only, depression and smoking risks, or smoking and IPV risks showed higher non-completion rates than women in the other risk groups.

**Table 5:** Comparison of non-completion of follow-up and postpartum interviews by African American participants by risk characteristics

**Risk characteristics at baseline interview**	**Number eligible & completed baseline**	Not completed a follow-up interview prior to delivery*		**Not completed the final postpartum interview**	
		
		N	%**	N	%**
**Active smoking:**					
Yes	198	43	21.7	46	23.2
No	846	151	17.8	167	19.7
**Environmental tobacco smoking (ETSE):**					
Yes	742	147	19.8	153	20.6
No	283	44	15.6	57	20.1
**Depression:**					
Yes	463	94	20.3	96	20.7
No	581	100	17.2	117	20.1
**Intimate partner violence (IPV):**					
Yes	336	68	20.2	62	18.5
No	708	126	17.8	151	21.3
**Risk groups:**					
Smoking (active & ETSE) only	325	59	18.2	66	20.3
Depression only	63	12	19.0	16	25.4
Intimate partner violence only	28	2	7.1	3	8.0
Smoking & depression	193	38	19.7	46	23.8
Smoking & intimate partner violence	101	22	21.8	25	24.8
Depression & partner violence	37	8	21.6	5	13.5
All risks	170	36	21.2	29	17.1
**Any of the 4 risks:**					
Yes	907	175	19.3	190	20.9
No	137	19	13.9	23	16.8
**Number of risks:**					
0	137	19	13.9	23	16.8
1	360	61	16.9	76	21.1
2	298	60	20.1	68	22.8
3	213	45	21.1	39	18.3
4	36	9	25.0	7	19.9
**Number of risks (exc. ETSE):**					
0	388	63	16.2	75	19.3
1	358	67	18.7	82	22.9
2	255	54	21.2	46	18.0
3	43	10	23.3	10	23.3
**Alcohol use during pregnancy:**					
Yes	223	37	16.6	43	19.3
No	820	157	19.1	170	20.7
**Drug use during pregnancy:**					
Yes	181	42	23.2	33	18.2
No	863	152	17.6	180	20.9
**Care group:**					
Intervention	521	98	18.8	114	21.9
Usual Care	523	96	18.4	99	18.9
**Total**	1044	194	18.6	213	20.4

* Completed the second follow-up interview prior to delivery or the first follow-up interview and delivered prior to the time to administer the second follow-up interview.

** Percentages are calculated of the total number of eligible women who completed the baseline interview (second column).

Moreover, women reporting no specific risk factor at baseline were less likely to drop out from the study than those with risk, perhaps because of less pressure on attending intervention sessions and fewer demands on their time. Randomized group assignment (to either intervention or usual care group) did not appear related to women's completion rates, suggesting that reasons for attrition and dropout from the study were not intervention-specific.

### Multivariable comparison of characteristics of participants who completed the study

A multivariable analysis was performed in which we compared women who were retained until the end of the study and for whom the postpartum final interview was completed, to those who were lost to follow-up or dropped out and whose postpartum final interview was not completed. Table 6 presents the adjusted odds ratios (AOR) of the logistic regression analysis for African American participants not retained and thus not completing the postpartum final interview. The results of the proposed model indicated lower retention rates were predicted for women who were young (< 28 years of age), less educated, single, separated, widowed, or divorced, not working, with at least four of her own children less than 18 years of age living at home with her, receiving Medicaid, not receiving WIC, not alcohol or drug users, active smokers or exposed to ETSE, not depressed, or had experienced IPV.

**Table 6:** Logistic regression analysis on African American participants not completing the postpartum final interview

**Characteristics at baseline interview**	AOR* (95% CI)
**Maternal age:**	
18–22	1.38 (0.87,2.20)
23–27	1.41 (0.89,2.42)
28 +	1.00
**Educational level:**	
< High school	1.67 (0.98,2.85)
HS graduate/GED	1.37 (0.84,2.21)
At least some college	1.00
**Relationship status:**	
Single/separate/widowed/divorced	1.54 (1.00,2.36)
Married or living with partner	1.00
**Current work status:**	
Working (full- or part-time)	0.84 (0.58,1.22)
Not currently working	1.00
**No. children < 18 living at home:**	
< 4 children	0.50 (0.26,0.96)
4+ Children	1.00
**Medicaid status:**	
Yes	1.11 (0.70,1.78)
No	1.00
**WIC status:**	
Yes	0.14 (0.10,0.21)
No	1.00
**Alcohol use during pregnancy:**	
Yes	0.96 (0.62,1.48)
No	1.000
**Drug use during pregnancy:**	
Yes	0.77 (0.48,1.24)
No	1.00
**Clinic site:**	
A	0.58 (0.34,0.98)
B	2.22 (1.29,3.81)
C	0.42 (0.24,0.73)
D	0.72 (0.43,1.21)
E	0.81 (0.44,1.50)
F	1.00
**Active smoking:**	
Yes	1.17 (0.75,1.82)
No	1.00
**Environmental tobacco smoking (ETSE):**	
Yes	1.03 (0.70,1.52)
No	1.00
**Depression:**	
Yes	0.99 (0.70,1.40)
No	1.00
**Intimate partner violence (IPV):**	
Yes	0.70 (0.48,1.03)
No	1.00

* AOR is adjusted odds ratio.

However, only a few of these characteristics were statistically significant. The most significant variable, controlling for site differentials, was WIC. Pregnant women who did not receive WIC were 86% more likely not to complete the postpartum final interview (AOR = 0.14, 95% CI: 0.10 – 0.21). Number of children less than 18 years of age living at home with their mother also significantly predicted retention status. Mothers with fewer than 4 children at home were less likely to complete the postpartum interview (AOR = 0.50, 95% CI: 0.26 – 0.96). Single, separated, widowed, or divorced women were more likely not to complete the postpartum interview (AOR = 1.54, 95% CI: 1.00 – 2.36).

Education level was not significant in this model. Women with less than high school education were more likely not to complete the postpartum interview (AOR = 1.67, 95% CI: 0.98 – 2.85). This finding may be due to the differentials in the education levels across clinic sites and presence of risk factors in the model. The Chi-square test for cross tabulation of clinic sites by education level indicated that education levels were statistically different across sites. Similarly, education level was statistically different with respect to presence or absence of active smoking risk, ETSE risk, or depression risk. Other socio-demographic and risk characteristics were not significant in predicting the completion of the postpartum interview.

### Identification of subgroups of women with low or high rates of completion of the study

The Classification and Regression Trees (CART) procedure was used to identify the characteristics of those homogeneous subgroups of African American women who were less (more) likely not to be retained at postpartum. Identification of these subgroups can aid investigators who are conducting research on minority populations to focus retention strategies toward those subgroups of women who could have low retention rates.

**Figure 2:**
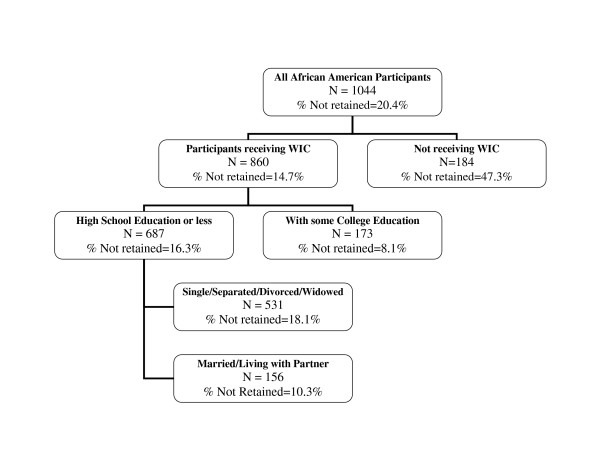
CART Analysis of Retention Rates by Various Characteristics.

The CART methodology was implemented using all the predictors shown in Table 6. For these categorical characteristics, there are over 110 thousand possible combinations. Table 7 presents the results of running the CART procedure with various subsets of these predictors and displays the socio-demographic and risk combinations of African American women not completing the postpartum interview. While the overall non-retention (non-completion of postpartum interview) rate for all African American women was 20%, various subgroups of women experienced higher or lower retention rates. In the first panel, two subgroups of women were identified with very high rates of non-completion of the postpartum interview (> 40%). In the second panel, four subgroups of women were identified with high rates of non-completion (30–40%), while six subgroups of women in the third panel were identified with very low rates of non-completion of the postpartum interview (< 10%). Figure 2 shows an example of these subgroups with different retention rates, as created by the CART-based classification tree procedure.

**Table 7:** Characteristics of African American pregnant women by rates of not completing the postpartum final interview

**Subgroup**	**Socio-demographic characteristics^# ^of African American pregnant women**	**% not completing postpartum interview**	**N**
**1. Very high rates of non completion (> 40%)**			
1.1	Not receiving WIC	47.3	184
1.2	Single, HS or less, drug user, 23–27 years old	40.7	27
**2. High rates of non completion (30–40%)**			
2.1	< HS, drug user, < 28 years of age, alcohol user	38.1	21
2.2	Not working, drug user, 23–27 years of age	36.4	33
2.3	< 28 years old, alcohol user, receiving Medicaid	32.1	106
2.4	With 4+ children < 18 years living at home	31.3	67
**3. Low rates of non completion (< 10%)**			
3.1	With < 4 children below 18 years living at home, 28+ years, alcohol user	5.4	74
3.2	< 28 years, alcohol user, no Medicaid	5.7	35
3.3	WIC recipient, HS or less, single, drug user, 18–22 or 28+ years	7.1	70
3.4	WIC recipient, at least some college	8.1	173
3.5	WIC recipient, less than HS, single, not drug user, Medicaid recipient	8.3	12
3.6	28+ years, alcohol user	8.5	82

# Socio-demographic characteristics are those identified at baseline interview.

As shown in Table 7, the characteristics of the two subgroups of women with *the highest rates of non-retention *(or highest rates of not completing the postpartum interview) are: (1) women not receiving WIC (47.3% not retained for the final interview; see Figure 2), and (2) single women, 23–27 year olds with less than high school education who were drug users (40.7% not retained).

The second panel of subgroups of women is those with *30–40% non-retention rates*. Four such subgroups are shown in Table 7. Examples of these subgroups are:

(1) women with less than high school education, young (< 28 years old), and women using alcohol and drugs (38.1% were not retained until the end of the study),

(2) women 23–27 years old who were not working and were drug users had a low retention rate (36.4% were not retained),

(3) young women (< 28 years old) who were alcohol users and receiving Medicaid (32.1% were not retained),

(4) women with at least four children below 18 years of age living at home (31.3% were not retained).

The third panel of subgroups of women are those with the *lowest rates of non-retention*. Examples of these subgroups are:

(1) older women (at least 28 years of age), alcohol users, and with less than four children aged less than 18 years living at home (high retention rate at the end of the study: only 5.4% did not complete the final interview),

(2) alcohol users less than 28 years old and not receiving Medicaid (low rate of not completing the final interview of 5.7%),

(3) WIC recipients who are single 18–22 or 28+ years old with high school education or less and single (non-retention rate of 7.1%),

(4) WIC recipients with some college (non-retention rate of only 8.1%, as shown in Figure 2),

(5) WIC and Medicaid recipients with less than high school who were single and not using drugs (8.3% non-retention rate),

(6) women 23–27 years old with at least high school education who were not depressed, experienced no IPV, and receiving no Medicaid (high retention rates: 9.5% did not complete the final interview).

From the above lists, it is evident that single, less educated, drug and alcohol using, and non-working women and WIC non-recipients may represent minority women with expected high attrition rates. These subgroups of women identified with very high or high non-retention rates may deserve special attention and additional efforts in future research on low-income minority women, in order to achieve higher rates of retention and to increase the generalizability of research results.

## Discussion

The successful recruitment and retention of low-income minority women in Project DC-HOPE is in marked contrast to previous reports for similar ethnic minorities in clinical research [3,11,12,31-33]. It has been reported that recruitment rates of ethnic minorities in clinical trials are small when compared to non-minorities [31,34,35]. Our results can, to some degree, be attributed to the resources expended. As Powe and Gary [12] have suggested, incentives play an important role in the initial recruitment and subsequent retention of this ethnic minority population. The project supported a clinic site administrator to ensure the integration of the project into the clinic's everyday activities. In addition, the project design contributed to our recruitment and retention success. This included enrolling participants after they completed the baseline interview and scheduling intervention sessions to coincide with prenatal care visits. Also, the subject matter and timing of this study likely contributed to the successful recruitment and retention of participants. Pregnancy is a finite time interval during which the health of the baby is of heightened concern. Additionally, mothers may be more motivated to make behavior changes to improve their own health as well as that of their unborn infant.

These strategies alone were not likely sufficient, however. A great contribution to our success was the conscientious and dedicated efforts of the project teams. They effectively used the DMS and skillfully applied the strategies taught in training. Matching the RSs and PAs to our study population in terms of gender and ethnicity was also critical, as has been demonstrated in other research efforts. Moreno-John, et al. [36] report that the Resource Centers on Minority Aging Research employ staff and researchers that are ethnically and culturally matched to community participants, since it has been found that employment of researchers and staff reflective of the community improves subject satisfaction and adherence. Similarly, using interviewers of the same ethnic backgrounds as their subjects [3] and employing project staff who pay attention to cultural sensitivity [37-39] enhance recruitment and retention of minority populations. The collection of multiple telephone numbers, sharing updated contact information, and effective tracing techniques also led to high retention. Monitoring study progress to identify troubleshooting strategies, close supervision by the project management team, and convening meetings to discuss issues and resolve problems provided a research environment that was cohesive and goal-oriented.

Of all eligible minority women, 85% agreed to participate. Of those agreeing, 90% completed the baseline interview and were enrolled. This finding is counter to the assertion that African-American and minority women are unwilling to participate in research. In Project DC-HOPE, over 60% of those who refused to participate did so because they denied the need for help or lacked interest, which is conceptually similar to reasons reported in the literature [12] but contrary to the themes of lack of trust or fear. We speculate that the significant differences in enrollment rates by clinic site were a function of the recruitment skills of the RSs and the predisposition of the potential participants.

The lack of substantial differences in the socio-demographic characteristics and risk profiles of women retained and not retained in the study indicates similarity between the two groups, and suggests that this factor did not bias the findings of the study. Randomization into the intervention or the usual care group did not impact significantly on women's retention rates. Thus, reasons for non-retention were not intervention-specific. However, the differences between women who were lost to follow-up and women who dropped out of the study are interesting to note. Women who dropped out were more educated, more likely to be married and employed, older, and less likely to be receiving Medicaid.

In addition, women not receiving WIC were less likely to complete the postpartum interview compared to women who were receiving WIC. A profile of the WIC children [40] shows them to be economically needier than the non-WIC children, and the average age of their mothers at the time of birth was 25 years, with 8% under the age of 18. Our results show that the WIC women were significantly less educated, not working, and more likely to be receiving Medicaid than the non-WIC women. The WIC women were younger than the non-WIC women (24.4 vs. 25.2 years). It is possible that women who dropped out of the study had more stable lives and viewed the study as less beneficial to them. For these women, the benefits of the study must consistently be reinforced throughout the duration of the study to prevent them from losing interest and dropping out. Interestingly, women with only IPV risk had the lowest non-completion rates of any risk group, perhaps finding the study beneficial to them.

On the other hand, women who were lost to follow-up were less educated, not married, unemployed, younger and receiving Medicaid. In our prediction models, being single, separated, widowed, or divorced was a significant predictor for not completing the postpartum interview. Although not significant, prediction results indicated a trend for women with less than high school education being more likely not to complete the postpartum interview. Even though the WIC recipients had higher interview completion rates than the non-WIC recipients, among women who were lost to follow-up, 65% were receiving WIC as compared to 38% among women who dropped out. In addition, women in the usual care group, with whom there was less contact throughout the study, were also more likely to be lost to follow-up as compared to the intervention group. We conclude that the women lost to follow-up were likely less stable and more difficult to contact and retain. Those women are young, less educated, less likely to be married or employed, and more likely to be on Medicaid. For such women, investigators should emphasize the need for obtaining accurate and complete contact information in order to be able to minimize non-retention as much as possible.

## Conclusion

The success of the recruitment and retention efforts in Project DC-HOPE indicates that difficulties in recruiting and retaining minorities for clinical and behavioral research are not insurmountable. With targeted recruitment and retention strategies, minorities will participate at high rates in these trials. It is important that they be included in these trials because the prevalence of many health problems is higher in ethnic minorities, and health outcomes are often poorer. Consequently, the reduction of racial and ethnic health disparities has become an important public health objective, requiring concerted and innovative efforts. In addition, the inclusion of ethnic minorities in clinical and behavioral research provides better access to new and high-quality health care often not available to them, and permits the study of potential ethnic differences in the pathology of disease. Finally, increased participation of ethnic minorities will result in improved generalizability of research results.

## Abbreviations

A-CASI – Audio-Computer-Assisted Self Interview

AOR – Adjusted Odds Ratios

CART – Classification and Regression Trees

DCC – Data Coordinating Center

DMS – Data Management System

DC-HOPE – intervention trial entitled "Interventions for Risk Factors in Pregnant Women in Washington, DC: An Integrated Approach" or DC-Healthy Outcomes of Pregnancy Education

ETSE – Environmental Tobacco Smoke Exposure

IPV – Intimate Partner Violence

RS – Recruitment Specialists

PA – Pregnancy Advisors/Interventionists

WIC – Women, Infants and Children's Program

RTI International – A trade name of Research Triangle Institute

## Competing interests

The author(s) declare that they have no competing interests.

## Authors' contributions

M. Nabil El-Khorazaty:

MNE, as the P.I. of the DCC, made substantive contributions to the conception, planning, design, sample size determination, development of the instruments, development of the Data Management System (DMS), and conduct of the study. He also monitored recruitment, data collection, and follow-up activities, supervised data processing, developed the analysis plan, conducted interim analysis and reported to the Data Safety Monitoring Board (DSMB), performed substantial statistical analyses, participated in the writing of the manuscript, and finally MNE has given final approval of the manuscript for publication.

Allan A. Johnson:

AAJ had extensive involvement in the planning of recruitment and retention procedures and documents and provided help in the developments of instruments and selection and provision of orientations to clinic sites. AAJ also participated in selection, training, assignment of recruitment specialists, and meeting with them. AAJ participated in data analysis activities and interpretation of the results, and drafting and review of the manuscript, and he has given final approval of the version to be published.

Michele Kiely:

MK, as the NICHD Project Officer, oversaw all the activities of the study while it was in the field. She participated in the analysis and interpretation of the results. MK did a significant amount of the original writing of the manuscript, as well as revising it critically for important intellectual content; finally, MK has given final approval of the version to be published.

Ayman A.E. El-Mohandes:

AAE was directly involved in the design and implementation of all aspects of this study. He monitored all activities related to recruitment and retention as the Executive P.I. of the NIH-DC Initiative to Reduce Infant Mortality in Minority Populations. AAE also participated directly in the analysis plan and the interpretation of results. He participated in the authorship of the paper; he reviewed and edited, when necessary, and approved the text as it is presented in its final form.

Siva Subramanian:

SS made significant contributions beginning from the conception and design of this study. He also participated in the analysis and interpretation of data. SS was also involved in reviewing the manuscript and contributed to the improvement of the manuscript. SS approves the final version of this manuscript.

Haziel A. Laryea:

HAL helped in the planning of recruitment activities, development of field instruments and forms, providing site orientation and training of recruitment specialists. HAL had extensive involvement in the implementation and supervision of recruitment and retention activities including assignment of recruitment specialists, participation in regular meetings, and monitoring their day-to-day activities.

Kennan B. Murray:

KBM contributed to the planning and design of the study, development of the study instruments, training of data collectors, developing of the DMS, help in the development of manuals of operations, monitoring field activities, prepared monitoring and tracking reports of all recruitment, interviewing, and follow-up activities, contributed to the interim analyses, performed the statistical analysis, contributed to the draft of the manuscript, provided substantial interpretation of data, and KBM gave final approval of the manuscript for publication.

Jutta S. Thornberry:

JST contributed to the planning and design of the study, development of the study instruments, development of the operations manuals, training of data collectors, monitoring field activities. She also contributed to the draft of the manuscript, provided substantial interpretation of data and results, and JST gave final approval of the manuscript for publication.

Jill G. Joseph:

JGJ made substantial contributions to conception and design of this study and to analysis and interpretation of data; she has also been involved in drafting the manuscript and revising it critically for important intellectual content; finally, JGJ has given final approval of the version to be published.

## Pre-publication history

The pre-publication history for this paper can be accessed here:


